# Health Surveillance and Response to SARS-CoV-2 Mass Testing in Health Workers of a Large Italian Hospital in Verona, Veneto

**DOI:** 10.3390/ijerph17145104

**Published:** 2020-07-15

**Authors:** Stefano Porru, Angela Carta, Maria Grazia Lourdes Monaco, Giuseppe Verlato, Andrea Battaggia, Marco Parpaiola, Giuliana Lo Cascio, Manuela Pegoraro, Valentina Militello, Francesca Moretti, Stefano Tardivo

**Affiliations:** 1 Section of Occupational Medicine, Department of Diagnostics and Public Health, University of Verona, 37134 Verona VR, Italy; stefano.porru@univr.it; 2 Clinical Unit of Occupational Medicine, University Hospital of Verona, 37134 Verona VR, Italy; mariagrazialourdes.monaco@aovr.veneto.it; 3 Unit of Epidemiology and Medical Statistics, Department of Diagnostics and Public Health, University of Verona, 37134 Verona VR, Italy; giuseppe.verlato@univr.it; 4 Postgraduate School of Occupational Medicine, University of Verona, 37134 Verona VR, Italy; andrea.battaggia@studenti.univr.it (A.B.); marco.parpaiola@studenti.univr.it (M.P.); 5 Unit of Microbiology and Virology, University Hospital of Verona, 37134 Verona VR, Italy; giuliana.locascio@aovr.veneto.it (G.L.C.); manuela.pegoraro@aovr.veneto.it (M.P.); valentina.militello@aovr.veneto.it (V.M.); 6 Section of Hygiene, Department of Diagnostics and Public Health, University of Verona and Medical Direction, University Hospital of Verona, 37134 Verona VR, Italy; francesca.moretti76@gmail.com (F.M.); stefano.tardivo@univr.it (S.T.)

**Keywords:** mass screening, healthcare workers, COVID-19, swab test

## Abstract

Italy presented the first largest COVID-19 outbreak outside of China. Veneto currently ranks fourth among the Italian regions for COVID-19 confirmed cases (~19,000). This study presents health surveillance data for SARS-CoV-2 in 6100 health workers (HW) employed in a large public hospital. Workers underwent oropharyngeal and nasopharyngeal swabs, with a total of 5942 participants (97.5% of the population). A total of 11,890 specimens were tested for SARS-CoV-2 infection using PCR, identifying the viral genes E, RdRP, and N. Positive tests were returned for 238 workers (cumulative incidence of 4.0%, similar in both COVID and nonCOVID units). SARS-CoV-2 risk was not affected by gender, age, or job type, whereas work setting and occupation were both predictors of infection. The risk was higher in medical wards (OR 2.7, 95% CI 1.9–3.9) and health services (OR 4.3, 95% CI 2.4–7.6), and lower in surgical wards and administration areas. To our knowledge, this study represents the largest available HW case list swab-tested for SARS-CoV-2, covering almost the total workforce. Mass screening enabled the isolation of HW, improved risk assessment, allowed for close contacts of and infected HW to return to work, provided evidence of SARS-CoV-2 diffusion, and presented solid ground to prevent nosocomial SARS-CoV-2 infections. The ongoing concurrent sero-epidemiological study aims to enable the improvement of health surveillance to maintain the safety of HWs and the communities they serve.

## 1. Introduction

Italy presented the first largest COVID-19 outbreak outside of China and currently ranks sixth among countries for COVID-19 confirmed cases [1]. According to the Italian National Health Authority, Istituto Superiore di Sanità (ISS), 225,549 COVID-19 cases and 30,332 associated deaths have occurred as of 18 May [2].

The Veneto region is in Northeastern Italy and currently ranks fourth among Italian regions for COVID-19 confirmed cases (*n* = 18,958), behind Lombardy (84,844), Piedmont (29,968), and Emilia Romagna (27,232) [2].

Veneto was first involved in the infection chain during February, with the well-known first cluster occurring in a small village and the first death occurring on 21 February [3]. After this, cases increased sharply. Veneto introduced restrictions inspired by previous experiences [4,5,6], including isolation, contact tracing and mass testing, and a focus on nosocomial transmission, in accordance with international guidelines [7,8].

This strategy impacted the progression of the COVID-19 epidemic in Veneto in a different way compared to other high prevalence Italian regions [9,10]. The most recent regional data showed a significant improvement of the epidemic curve with reduced new cases, decreased intensive care unit patients, and increased recoveries [11].

Healthcare settings are workplaces at high risk for COVID-19, and health workers (HW) are likely to acquire SARS-CoV-2 as an occupational infection; at the same time, they may transmit SARS-CoV-2 to vulnerable patients, other HW, or visitors [12,13,14,15].

A remarkable toll on HW has already been caused by COVID-19. Different strategies regarding lockdown, reconstruction of transmission chains, health surveillance (HS), and monitoring of HW were adopted according to specific geographical areas, local epidemics, logistic considerations, and availability of diagnostic tests, with different preventive outcomes [16,17,18,19,20,21,22,23].

In Italy, 26,426 COVID-19 cases were reported among HW, i.e., ~11.7% of all Italian confirmed cases [2].

To our knowledge, this is the first study aiming to report HS data for SARS-CoV-2, carried out with updated mass diagnostic nasal and oropharyngeal swab tests and involving a very large cohort of symptomatic and asymptomatic HW. These HW belonged to a large public university hospital located within a critical geographical area, with an almost complete coverage of the HW workforce. The secondary aim of this research was to learn lessons regarding risk assessment, HS, and other preventive strategies.

## 2. Materials and Methods

### 2.1. Population and Settings

The University Hospital of Verona is the second largest hospital trust in Italy in terms of bed number and the fifth largest in terms of admissions. It currently employs about 6100 HW, including about 1150 resident physicians, deployed in two facilities (Ospedale Maggiore and Policlinico G.B.Rossi) located in opposite areas of the city of Verona.

This study included all employees (patient care, technicians, and administrative staff) and residents under contract working at the hospital during the onset of the epidemic.

We excluded workers on parental or sick leave, contractors (because they were not under the formal responsibility of surveillance by the University Hospital, nor they were included in the health surveillance programs established by the Regional Health Authority), and a few residents, undergraduate, and postgraduate trainees not currently working at the hospital.

The hospital possesses 1215 beds with 124 day beds and hosts a range of medical and surgical wards, emergency departments, infectious disease divisions, and intensive care units. It serves a provincial area with 922,000 inhabitants and patients recruited from various regions.

The first COVID-19 patient and HW cases were respectively admitted on 26 February 2020 and 29 February 2020.

### 2.2. General Organization

At the onset of the epidemic, five areas were identified as COVID-19 units, plus two emergency rooms. Two wards were included as high-risk areas based on the cluster of cases that subsequently emerged.

Various preventive measures were adopted, such as controlled access, personal protective equipment (PPE) according to the World Health Organizations (WHO,) ISS and regional recommendations, isolation measures, training, specific biohazard procedures, source control, contact tracing, and testing.

On 23 February, Verona Hospital deployed a Crisis Unit, including general, medical, and nurse managers, directors of emergency, infectious diseases, and intensive care departments, plus hygiene and occupational medicine units, to implement multiple concurrent preventive interventions. Trade unions and safety representatives were constantly involved. Newsletters were sent to all HW to organize surveillance and swab tests.

### 2.3. Risk Assessment

Occupational risk assessment was carried out according to Italian Guidelines for Biological Occupational Risk and CDC guidance [24,25]. The four risk categories were high, medium, low, and not identifiable.

### 2.4. Personal Protective Equipment 

Internal guidelines for mandatory use of PPE were adopted based on WHO and ISS guidelines [26,27].

### 2.5. Health Surveillance 

Measures were based on national and regional procedures and updated according to epidemiological changes. Until the middle of March 2020, self-isolation and self-surveillance of body temperature and respiratory symptoms for 14 days was required for all close contact. Definition of close contact included a person with direct physical contact with a COVID-19 case (e.g., shaking hands), a person with unprotected direct contact with infectious secretions of a COVID-19 case (e.g., touching used tissues with bare hands), a person with face-to-face contact with a COVID-19 case within 2 m for over 15 min, a person who was in a closed environment (e.g., classroom, meeting room, waiting room) with a COVID-19 case within 2 m for over 15 min, a HW providing direct care for a COVID-19 case, and laboratory workers handling specimens from a COVID-19 case without recommended PPE or with a possible breach of PPE.

To tackle the possible HW shortage and face the epidemic curve, these procedures were re-evaluated; the last version was issued on 28 April by the Health Authority. HS included HW monitoring by repeated swabs and, recently, by serological tests, with specific schedules according to risk indicators, such as being a close contact of a COVID-19 case and working in a high-risk area (e.g., COVID units and emergency areas) [7].

A clinic dedicated to COVID-19 HS for HW was established on 9 March 2020 and was implemented along the way; at its maximum, 36 medical residents and 10 nurses were involved for 13 h a day, 7 days a week.

HS was integrated using organizational measures such as front and back offices to manage notifications to attend the clinic, communications, outcomes of HS, and isolation measures. Software and related databases were established ad hoc to include various personal, clinical, and epidemiological data and compensation aspects related to occupational injury or disease.

HW received emails to call for swab tests. Results were available 24–36 h after testing at the individual electronic dossier.

A mass screening strategy was adopted. Close contacts were the priority targets. Contact tracing was carried out through the coordination of the hospital Crisis Unit and the local Public Health Authority. Close contacts were those who had contact within 14 days prior to the contact reporting date. After 14 days from the date of close contact, if the HW did not show symptoms and if the swabs showed no detectable viral load, specific HS ended. All HW personnel were invited for mass screening swab tests regardless, which was conducted using different schedules according to the level of risk.

Clinical organization, contact tracing, and return-to-work procedures are herein reported in flow charts (see Supplementary Material).

Data were collected between 28 February and 28 April.

### 2.6. Oropharyngeal (OP) and Nasopharyngeal (NP) Swabs

For each HW, OP and NP (both nostrils) swabs were collected by trained medical personnel, according to ISS recommendations and CDC guidelines [28,29].

### 2.7. Real Time PCR

Respiratory specimens were tested for SARS-CoV-2 infection by commercial real-time PCR, Seegene AllplexTM2019-nCoV Assay (Seegene, Seoul, South Korea), according to manufacturer protocols. Automated RNA extraction and PCR set-up were carried out using Seegene NIMBUS, a liquid-handling workstation. Real-time PCRs were run on a CFX96TMDx platform (Bio-Rad Laboratories Inc., Hercules, CA, USA) and interpreted by Seegene’s Viewer software.

The Seegene assay identified the virus by a multiplex real-time PCR targeting three viral genes (E, RdRP, and N genes), complying with international testing protocols. Validation of the results was performed with the National Reference Laboratory of ISS, and the Seegene Assay was considered to be adequate for COVID-19 diagnosis [30]. The limit of detection for the assay was 4.8 copies/mL [31]. According to the manufacturer’s data, the positive percentage agreement with an RT-PCR comparator was 100% and the negative percentage agreement was 93.07%. Reported data on sensitivity and specificity were both 100%.

### 2.8. Ethics

The research was performed following the 1964 Declaration of Helsinki standards and its later amendments, and was launched and approved by the Institutional Board of the Regional Health Authority [7].

### 2.9. Statistical Analysis

The Clopper–Pearson method was used to calculate the 95% confidence interval (CI) of cumulative incidence of SARS-CoV-2 infection. Significance of differences among groups was evaluated by Fisher’s exact test or the chi-square test for categorical variables, and by one-way ANOVA or the Kruskal–Wallis test for quantitative variables, as appropriate. Multivariable analysis was accomplished using a logistic regression model, where SARS-CoV-2 detection was the response variable, and sex, age, work setting, and occupation were the potential determinants. Goodness-of-fit was verified by the Hosmer–Lemeshow test. Results were synthesized through the odds ratio (OR). The effect size was considered small, medium, large, or very large when the OR was approximately 1.5, 2.5, 4, or 10, respectively [32].

## 3. Results

### 3.1. Health Surveillance

On the basis of regional and hospital rules and directions, the Occupational Health Unit of the Verona University Hospital started HS for COVID-19 infection on 24 February 2020 for all 6092 employees working during the onset of the epidemic.

### 3.2. Participation

A total of 150 workers were not able to participate due to not answering requests for contact, despite being contacted at least three times. We hypothesized that they were either off work for various reasons (vacation, pregnancy, family matters, or sick leave) or that they were not able to see the emails.

The final number of participants was 5942 (97.5% of the target population). Table 1 reports the general features of the case list.

Gender proportion did not change as a function of participation; most workers were women, both among participating (69.1%) and nonparticipating (62.7%) subjects.

Nonparticipating HW were slightly younger (mean age ± SD = 40.0 ± 12.4 years, range: 24.4–66.9) than participating HW (44.3 ± 11.7 years, range: 22.3–70.4) (*p* < 0.001). Among nonparticipating HW, medical wards and religious assistance were overrepresented, while health services, including all hospital outpatient services (e.g., emergency rooms, diagnostic services, occupational medicine, forensic medicine, technical services) and hospital administration were underrepresented compared to participants.

Nearly all nurses, aides, physicians, other health professionals, and technical assistants took part in the survey; participation was lower among medical residents and administrative staff.

Only eight HW who were close contacts did not respond to multiple invitations to attend the swab clinic.

### 3.3. Cumulative Incidence of SARS-CoV-2 Infection

Positive swabs were obtained for 238 of 5942 workers, yielding a cumulative incidence of 4.0% (95% CI 3.5–4.5%). Workers infected by SARS-CoV-2 underwent a median number of five swabs (p25–p75 = 4–7); negative workers underwent a median number of one swab (p25–p75 = 1–2). Nearly all SARS-CoV-2 infections (n = 212) were found in workers undergoing swabs after close contact tracing; only 26 cases were detected within the ongoing mass screening. Accordingly, the proportion of positive cases was <1% in people not exposed to COVID-19 cases and 10% in exposed people. The presence of symptoms (such as fever, cough, dyspnea, sore throat, rhinoconjunctivitis, ageusia, and anosmia) was highly suggestive of SARS-CoV-2 infection, which was detected in nearly one-third of symptomatic subjects, but only in 2% of asymptomatic subjects (Table 2).

The incidence of SARS-CoV-2 infection peaked at 40.5% (117/289) in workers who were exposed to COVID-19 cases and presented suggestive symptoms, whereas the lowest incidence (0.5%) was detected in workers who neither were exposed nor presented symptoms.

The risk of SARS-CoV-2 detection significantly varied across work settings, observed to be the lowest in surgical wards and health services and the highest in medical wards and administrative units, including both administrative staff and public health physicians. Of note, the cumulative incidence of SARS-CoV-2 infection was about the same throughout COVID units and non-COVID units. On the other hand, the risk of SARS-CoV-2 detection was not significantly affected by gender, age, or type of job (Table 3).

In multivariable analysis, work setting and occupation emerged as significant predictors of SARS-Cov-2 infection. The risk of infection was observed to be highest in medical wards and health services and the lowest in surgical wards and hospital administration. Administrative staff were overall at lower risk of infection (Table 4). The corresponding effect size ranged from medium to large.

As of 29 April 2020, the total number of swabs performed was 11,890.

## 4. Discussion

So far, published reports on HS mostly focused on symptomatic HW, dealt with limited case lists and different selection criteria, or included HW from both hospital and territorial areas using different study designs, exposure patterns, and epidemiological backgrounds. Moreover, diagnostic procedures varied across the studies and even regarding time frames, therefore the results were possibly affected by different positive and negative predictive values.

We found no mass screening reported in the literature.

Wu et al. (2020) reported that HW represented a substantial proportion (3.8% = 1716/44,672) of all COVID-19 confirmed cases by 11 February in China [17].

Most studies performed in Western countries focused on HW with symptoms suggestive of COVID-19 infection. In early March 2020, only 45 (4.1%) out of 1097 symptomatic HW throughout nine hospitals in North Brabant, NL, were positive for SARS-CoV-2, with positive cases mostly concentrated in two settings [22]. In the second half of March, this percentage increased to 9.0% (112/1247) in a nearby university hospital [21]. A much higher prevalence of COVID-19 infection (18% = 282/1533) was found in symptomatic HW tested at a British teaching hospital during roughly the same period (16–29 March 2020) [20].

In a large hospital in Madrid, Spain, 2085 HW were tested, with 38% found to be infected, representing 11.6% of all HW [33]. In the Milan area, Northwestern Italy, 10% positive swabs were reported in the framework of contact tracing; no mass screening was carried out. Overall, 2.6% of positive cases were reported after “random testing” in HW [34].

Italian official reports showed that ~11.7% of all COVID-19 cases were diagnosed in HW.

We found a cumulative incidence of 4.0%, including both symptomatic and asymptomatic cases; when considering only those followed up for close-contact reasons, this proportion was 10%. As evident from data in the literature, and as expected from epidemic situations, the prevalence of positive HW varied significantly according to different settings and epidemiological conditions.

Our survey showed that SARS-CoV-2 infection in COVID units was almost the same as in other units, while work setting and occupation emerged as significant predictors of SARS-Cov-2 infection from a multivariable analysis. We presume that HW working in COVID units possessed greater knowledge and risk perception compared to other HW, which may have increased their compliance regarding preventive protocols (e.g., more attention paid to the wearing and removal of PPE, hand-washing procedures, etc.). Subsequently, the higher cumulative incidence of infection detected in medical wards and health services could be attributed to lower risk perception, especially at the beginning of epidemic, which may have significantly negatively influenced their adherence to preventive measured; moreover, transmission from asymptomatic subjects was possibly more likely occur to in medical areas.

The OR of infection was higher in health services and medical wards, probably because better compliance regarding preventive protocols (e.g., more proper use of PPE, increased hand hygiene, and diffusion of standard precautions) is achieved in high-risk areas, coupled with sound risk perception. Emergency room and diagnostic services were also both included in health services; personnel working in these services were probably exposed to mild symptomatic or asymptomatic patients in several unpredictable situations compared to ordinary activities.

The lower OR of infection in administrative staff confirmed that direct contact with patients represented the real risk. Almost all positive HW cases were close contacts of SARS-CoV-2 cases; being a close contact and presenting even mildly symptomatic therefore predicted positivity.

### Strengths and Weaknesses

To the best of our knowledge, this study is the first to report results of HS carried out with mass testing for SARS-CoV-2 in HW in a large public hospital in one of the highest risk Italian regions. The analyzed case list is also the largest compared to what we found in the literature.

In our opinion, the main advantages of our study were the mass screening carried out in a relatively short period of time, almost total coverage of the workforce, swab testing regardless of the presence of symptoms, a large number of swabs (both nasopharyngeal and oropharyngeal) with quality control of the diagnostic procedures, and overall organizational and pragmatic efforts involving collaboration between all parties.

This approach enabled the identification and prompt isolation of SARS-CoV-2-infected HW, allowing us to proceed with regular monitoring and limiting the risk of transmission of the virus, while minimizing danger for third parties and ensuring efficiency of the healthcare service itself. Moreover, risk assessment was constantly updated, allowing further interventions in specific wards, improvement of specific procedures, assessment of individual risk profiles, management of removal from workplaces and the return to work of close contacts and infected HW, and adjustment of swab schedules according to risk assessment.

On the other hand, we could not compare our data with other case lists due to different HS strategies, and we could not verify the real impact of specific preventive measures due to the study design. Future companion papers aim to report analyses and correlations with symptoms, use of PPE, origin of contact and infection, medicolegal issues, and other issues not tackled in this paper.

## 5. Conclusions

In conclusion, during an epidemic, an organized mass screening of all HW, regardless of presence of symptoms, provides not only a strong epidemiological evidence base regarding SARS-oV-2 infection among all HW, but also solid data on which to base focused interventions to prevent nosocomial SARS-CoV-2 infections and to monitor their effectiveness.

Moreover, the overall commitment of the management and all HW of Verona Hospital for COVID-19 prevention enabled the launch of a sero-epidemiological study, which is still ongoing, alongside swab screening, again with a very high participation rate.

The combined research aims to enable a better understanding of the epidemiology of SARS-CoV-2 infection, with the eventual goal of elaborating proper health surveillance within health care settings to ensure the safety of HW and the communities they serve.

## Figures and Tables

**Table 1:** Main demographic and occupational characteristics of workers participating or not participating in the survey.

	Participating Workers (*n* = 5942)		Nonparticipating Workers (150)		*p*-Value *
	*N*	%	N	%	
**Sex**					**0.107**
Men	1834	30.9	56	37.3	
Women	4108	69.1	96	62.7	
**Age (years)**					**<0.001**
22–29	1089	18.3	38	**25.3**	
30–39	1145	19.3	55	**36.7**	
40–49	1392	**23.4**	17	11.3	
50–59	1840	**31.0**	24	16.0	
60–70	476	8.0	16	10.7	
**Work setting**					**<0.001**
Medical ward	2014	33.6	58	**38.7**	
Surgical ward	1468	24.7	35	23.3	
Health services	1948	**32.8**	41	27.3	
Hospital administration	509	**8.6**	10	6.7	
Religious assistance	3	0.05	6	**4.0**	
**Working in COVID Unit**	1134	19.1	29	19.3	0.916
**Occupation**					**<0.001**
Physician	833	**14.0**	15	10.0	
Nurse	2022	**34.0**	12	8.0	
Other health professional	1041	17.5	18	12.0	
Resident	1083	18.2	65	**43.3**	
Technical assistant	395	**6.7**	3	2.0	
Administrative staff	495	8.3	17	**11.3**	
Other	73	1.2	20	**13.3**	
**Type of screening**					**<0.001**
Close contact of COVID-19 case	2123	**35.7**	8	5.3	
Screening program	3819	63.3	142	**94.7**	

* *p* values were computed by Fisher’s exact test or the chi-square test. Significant results are highlighted in bold.

**Table 2:** Factors affecting SARS-CoV-2 detection in 5942 workers of the University Hospital of Verona.

	*N*	Sars-CoV-2 Infected Subjects		*p*-Value
		N	%	
**Type of screening**				**<0.001**
COVID-19 cases	2123	**212**	**10.0**	
Screening program	3819	26	0.7	
**Symptoms**				**<0.001**
No	4695	109	2.3	
Yes	398	**124**	**31.2**	
Unknown	239	3	1.3	

*p*-values were computed using Fisher’s exact test. Significant results are highlighted in bold.

**Table 3:** COVID-19 cases in 5942 workers of Verona University Hospital, as a function of main demographic and occupational characteristics.

	*N*	COVID-19 Cases		*p*-Value
		N	%	
**Gender**				0.431
Men	1834	79	4.3%	
Women	4108	159	3.9%	
**Age (years)**				0.670
22–29	1089	51	4.7	
30–39	1145	46	4.0	
40–49	1392	50	3.6	
50–59	1840	75	4.1	
60–70	476	16	3.4	
**Work setting**				**<0.001**
Medical ward	2014	**125**	**6.2**	
Surgical ward	1468	*37*	2.5	
Health services	1948	*48*	2.5	
Hospital administration	509	**28**	**5.5**	
Religious assistance	3	0	0	
**Working in Covid.19 unit**				0.933
Yes	1134	46	4.1	
No	4808	192	4.0	
**Occupation**				0.254
Physician	833	32	3.8	
Nurse	2022	87	4.3	
Other health professional	1041	43	4.1	
Resident	1083	46	4.2	
Technical assistant	395	19	4.8	
Administrative staff	495	10	2.0	
Other	73	1	1.4	

*p*-values were computed using Fisher’s exact test. Significant results are highlighted in bold.

**Table 4:** Risk of COVID-19 infection in 5942 workers of Verona University Hospital, as a function of main demographic and occupational characteristics. Odds ratio (OR) with the corresponding 95% confidence intervals (95% CI) and *p* values were derived by a multivariable logistic model.

	OR (95% CI)	*p-*Value *
**Gender (Women vs. Men)**	0.85 (0.63–1.15)	0.297
**Age (years)**		0.492
22–29	1 (reference)	
30–39	0.89 (0.57–1.38)	
40–49	0.66 (0.40–1.08)	
50–59	0.76 (0.47–1-23)	
60–70	0.63 (0.32–1.24)	
**Work setting**		**<0.001**
Hospital administration	1 (reference)	
Medical ward	**2.74 (1.92–3.91)**	
Surgical ward	1.01 (0.65–1.58)	
Health services	**4.31 (2.44–7.62)**	
**Working in COVID-19 unit**	0.99 (0.70–1.40)	0.960
**Profession**		**<0.001**
Administrative staff/other	1 (reference)	
Physician	**3.61 (1.61–8.13)**	
Nurse	**4.15 (1.96–8.80)**	
Other health professional	**4.35 (2.05–9.23)**	
Resident	**2.95 (1.28–6.81)**	
Technical assistant	**4.04 (1.86–8.80)**	

* p-values were computed using likelihood ratios. Significant results are highlighted in bold.

## Supplementary Materials

The following are available online at https://www.mdpi.com/1660-4601/17/14/5104/s1, Figure S1: Flow chart for symptomatic health workers; Figure S2: Flow chart for asymptomatic health workers; Figure S3: Flow chart for indeterminate swabs.
