# Supplementary figures and images for: Health Surveillance and Response to SARS-CoV-2 Mass Testing in Health Workers of a Large Italian Hospital in Verona, Veneto
Source: Int J Environ Res Public Health. 2020 Jul 15;17(14):5104. doi: 10.3390/ijerph17145104 (PMC7399926; doi:10.3390/ijerph17145104)

**Figure 2. Flow chart for ASYMPTOMATIC Health Worker**

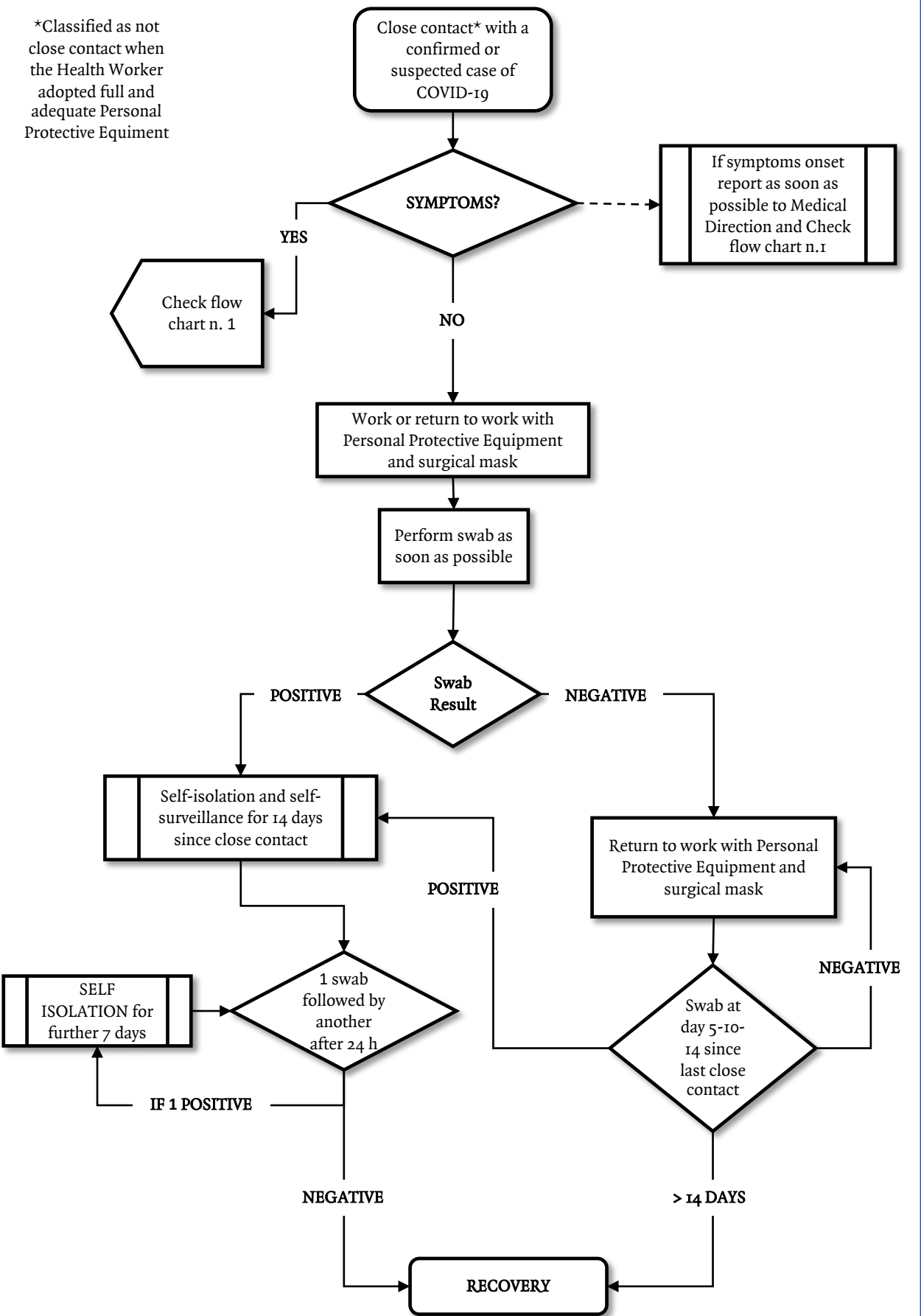

Supplement: Supplementary file 1 [file ijerph-17-05104-s001.zip › S2_Figure2.pdf]

**Figure 3. Flow chart for INDETERMINATE swab**

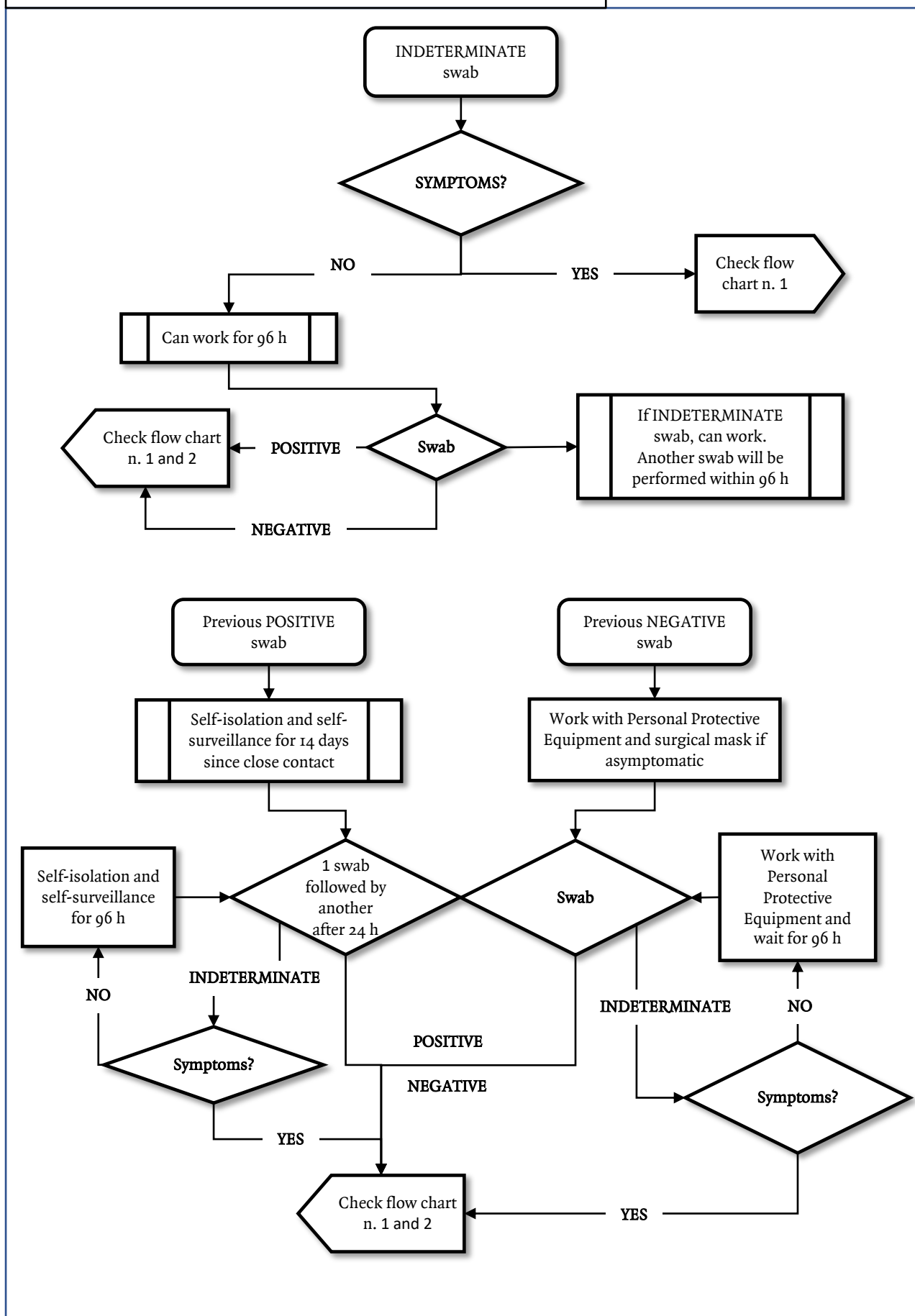

Supplement: Supplementary file 1 [file ijerph-17-05104-s001.zip › S3_Figure3.pdf]
